# Differential Susceptibility of Interneurons Expressing Neuropeptide Y or Parvalbumin in the Aged Hippocampus to Acute Seizure Activity

**DOI:** 10.1371/journal.pone.0024493

**Published:** 2011-09-06

**Authors:** Ramkumar Kuruba, Bharathi Hattiangady, Vipan K. Parihar, Bing Shuai, Ashok K. Shetty

**Affiliations:** 1 Medical Research and Surgery Services, Veterans Affairs Medical Center, Durham, North Carolina, United States of America; 2 Institute for Regenerative Medicine, Texas A&M Health Science Center at Scott & White, Temple, Texas, United States of America; 3 Research Service, Central Texas Veterans Health Care System, Temple, Texas, United States of America; 4 Division of Neurosurgery, Department of Surgery, Duke University Medical Center, Durham, North Carolina, United States of America; University of South Florida, United States of America

## Abstract

Acute seizure (AS) activity in old age has an increased predisposition for evolving into temporal lobe epilepsy (TLE). Furthermore, spontaneous seizures and cognitive dysfunction after AS activity are often intense in the aged population than in young adults. This could be due to an increased vulnerability of inhibitory interneurons in the aged hippocampus to AS activity. We investigated this issue by comparing the survival of hippocampal GABA-ergic interneurons that contain the neuropeptide Y (NPY) or the calcium binding protein parvalbumin (PV) between young adult (5-months old) and aged (22-months old) F344 rats at 12 days after three-hours of AS activity. Graded intraperitoneal injections of the kainic acid (KA) induced AS activity and a diazepam injection at 3 hours after the onset terminated AS-activity. Measurement of interneuron numbers in different hippocampal subfields revealed that NPY+ interneurons were relatively resistant to AS activity in the aged hippocampus in comparison to the young adult hippocampus. Whereas, PV+ interneurons were highly susceptible to AS activity in both age groups. However, as aging alone substantially depleted these populations, the aged hippocampus after three-hours of AS activity exhibited 48% reductions in NPY+ interneurons and 70% reductions in PV+ interneurons, in comparison to the young hippocampus after similar AS activity. Thus, AS activity-induced TLE in old age is associated with far fewer hippocampal NPY+ and PV+ interneuron numbers than AS-induced TLE in the young adult age. This discrepancy likely underlies the severe spontaneous seizures and cognitive dysfunction observed in the aged people after AS activity.

## Introduction

Epilepsy, characterized by intermittent and unpredictable occurrence of seizures, affects over 50 million people worldwide [1] and over two million people in the United States [2]. Furthermore, more than a third of epileptic patients are over 65 years of age, as old age is the most common time for presenting seizures [3]–[5]. This may be due to an increased excitability of principal hippocampal neurons seen with age [6]−[11]. From this perspective, the survival and connectivity of inhibitory interneurons in the aged hippocampus have received notice. Interneurons in the hippocampus are gamma-amino butyric acid (GABA) expressing non-principal neurons distributed in different strata of the dentate gyrus (DG), and the hippocampal CA1 and CA3 subfields. Inhibitory input from various subpopulations of GABA-ergic interneurons to principal neurons in different subfields of the hippocampus serves to keep the network stability [12]. Any disinhibition of the principal excitatory neurons due to compromised inhibitory input from the GABA-ergic interneurons leads to hyperexcitability [13]–[15]. Likewise, GABA agonists suppress seizures, GABA antagonists and drugs that inhibit GABA synthesis induce seizures, and drugs that increase synaptic GABA are potent anticonvulsants [16]. Thus, maintenance of a critical number of GABA-synthesizing interneurons in different subfields of the hippocampus appears essential for stabilizing excitatory influences and synchronizing principal excitatory neuron populations in the hippocampus [12], [15].

The interneuron population in the hippocampus is vulnerable to changes such as aging and excitotoxic hippocampal injury. Previous studies have demonstrated that aging leads to decreased numbers of GABA-ergic interneurons in all subfields of the hippocampus [17]–[22]. These findings indicate that decreased numbers of GABA-ergic interneurons have a role in the increased excitability of principal neurons in the aged hippocampus. A reduced functional inhibition observed in the aging hippocampus [23], [24] also supports the above possibility. Thus, circuitry in the aged hippocampus appears to be pro-excitatory and most vulnerable to conditions such as epilepsy. Indeed, the aged hippocampus exhibits an increased vulnerability to epileptic seizures after injury or exposure to excitotoxins [25]–[28]. Moreover, acute seizure (AS) activity in old age has an increased tendency for evolving into chronic temporal lobe epilepsy (TLE) [28]. Furthermore, spontaneous recurrent seizures (SRS) and cognitive dysfunction that ensue after AS activity are frequently intense in the aged population than in young adults [28]. Other studies have also shown an increased vulnerability of the aged population for developing TLE after brain damage resulting from stroke or head injury [29], [30]. In this context, an increased vulnerability of inhibitory GABA-ergic interneurons in the aged hippocampus to AS activity cannot be ruled out, though a recent study suggested resistance of GABA-ergic interneurons in the aged hippocampus to a focal excitotoxic injury [22].

To address the above issue, we compared the survival of subpopulations of hippocampal GABA-ergic interneurons that express the neuropeptide Y (NPY) or the calcium binding protein parvalbumin (PV) between the young adult (5-months old) and aged (22-months old) F344 rats following three-hours of AS activity. Graded intraperitoneal injections of the kainic acid (KA) were performed to generate AS activity, and AS activity was terminated using a diazepam injection at three hours after its onset. The NPY+ and PV+ interneurons in the dentate gyrus, and the CA1 and CA3 subfields of the young adult and aged hippocampi, were measured at 12 days after AS activity through a stereological method and compared with counts from the hippocampi of age-matched intact animals. The selection of NPY+ and PV+ interneurons for quantification in this study is based on the following reasons. The NPY is an endogenous anticonvulsant, and has a role in learning, memory and mood functions and hippocampal neurogenesis [31], [32]. Furthermore, the NPY+ interneurons in the dentate hilus play a crucial role in inhibiting the activity of hippocampal circuitry but more vulnerable to AS activity [33]. The PV+ interneurons contribute to synchronizing the hippocampal network oscillations [34]. Additionally, reductions in the PV+ interneuron subpopulation are thought to be a key factor in the epileptogenic process, as PV deficiency affects network properties resulting in an increased susceptibility to seizures [35], [36].

## Results

### Survival of NPY+ hippocampal interneurons following three-hours of AS activity

**Figure 1:**
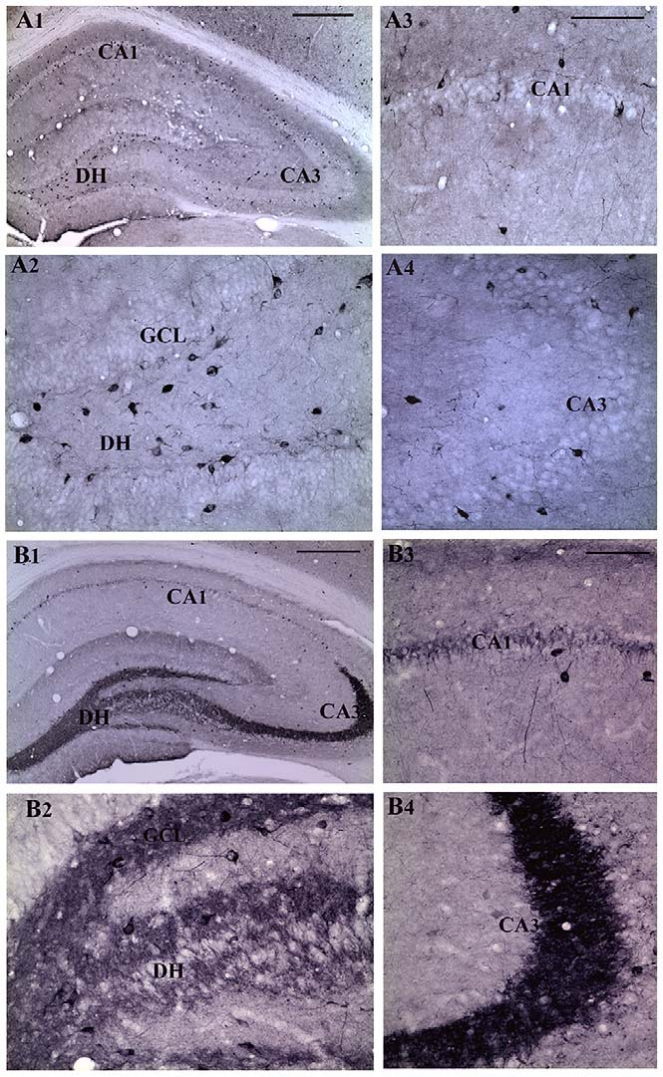
Distribution of neuropeptide Y-positive (NPY+) interneurons in different subfields of the hippocampus in an intact young adult rat (A1) and a young adult rat that underwent three hours of acute seizure (AS) activity (B1). Figures A2–A4 show magnified views of the dentate gyrus, the CA1 subfield and the CA3 subfield from the figure A1. Figures B2–B4 show magnified views of the dentate gyrus, the CA1 subfield and the CA3 subfield from the figure B1. Note that, three hours of AS activity induces a very robust NPY expression in the dentate mossy fibers of the young adult rat (B1, B2, and B4). Scale bar, A1 and B1 = 400 µm; A2–A4 and B2–B4 = 100 µm.

**Figure 2:**
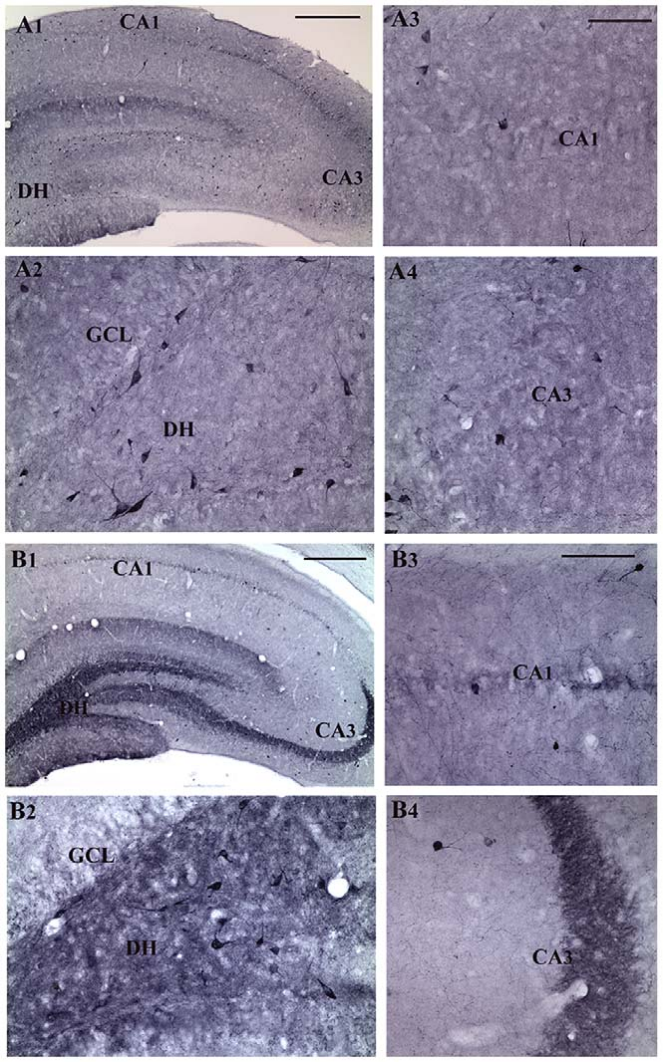
Distribution of neuropeptide Y-positive (NPY+) interneurons in different subfields of the hippocampus in an intact aged rat (A1) and an aged rat that underwent three hours of acute seizure (AS) activity (B1). Figures A2–A4 show magnified views of the dentate gyrus, the CA1 subfield and the CA3 subfield from the figure A1. Figures B2–B4 show magnified views of the dentate gyrus, the CA1 subfield and the CA3 subfield from the figure B1. Note that, three hours of AS activity induces NPY expression in the dentate mossy fibers of the aged rat (B1, B2, and B4). Scale bar, A1 and B1 = 400 µm; A2–A4 and B2–B4 = 100 µm.

Immunohistochemical staining of sections with a NPY antibody clearly demonstrated the prevalence of NPY+ interneurons in the dentate gyrus and the CA1 and CA3 subfields of both young and aged intact control rats (Figs 1 and 2 [A1]). The NPY+ neurons were prominent in the hilus of the dentate gyrus (Figs. 1 and 2 [A2]) and the strata oriens and pyramidale of the CA1 and CA3 regions (Figs. 1 and 2 [A3–A4]). The density of NPY+ interneurons in all subfields appeared greater in young rats (Fig. 1 [A2–A4] than in aged rats (Fig. 2 [A2–A4]), which is consistent with the previous reports [18], [38]. In comparison to the age-matched intact hippocampus, three hours of AS activity substantially reduced the density of NPY+ interneurons in different regions of the young hippocampus (Fig. 1 [B1–B4]), but not in the aged hippocampus (Fig. 2 [B1–B4]). However, both young and aged rats displayed an increased expression of NPY in the dentate mossy fibers within the hilus as well as in the principal mossy fiber bundle following three hours of AS activity (Figs. 1 and 2 [B1, B2, B4]), which is consistent with the purported role of the NPY in seizure modulation [32], [49].

**Figure 3:**
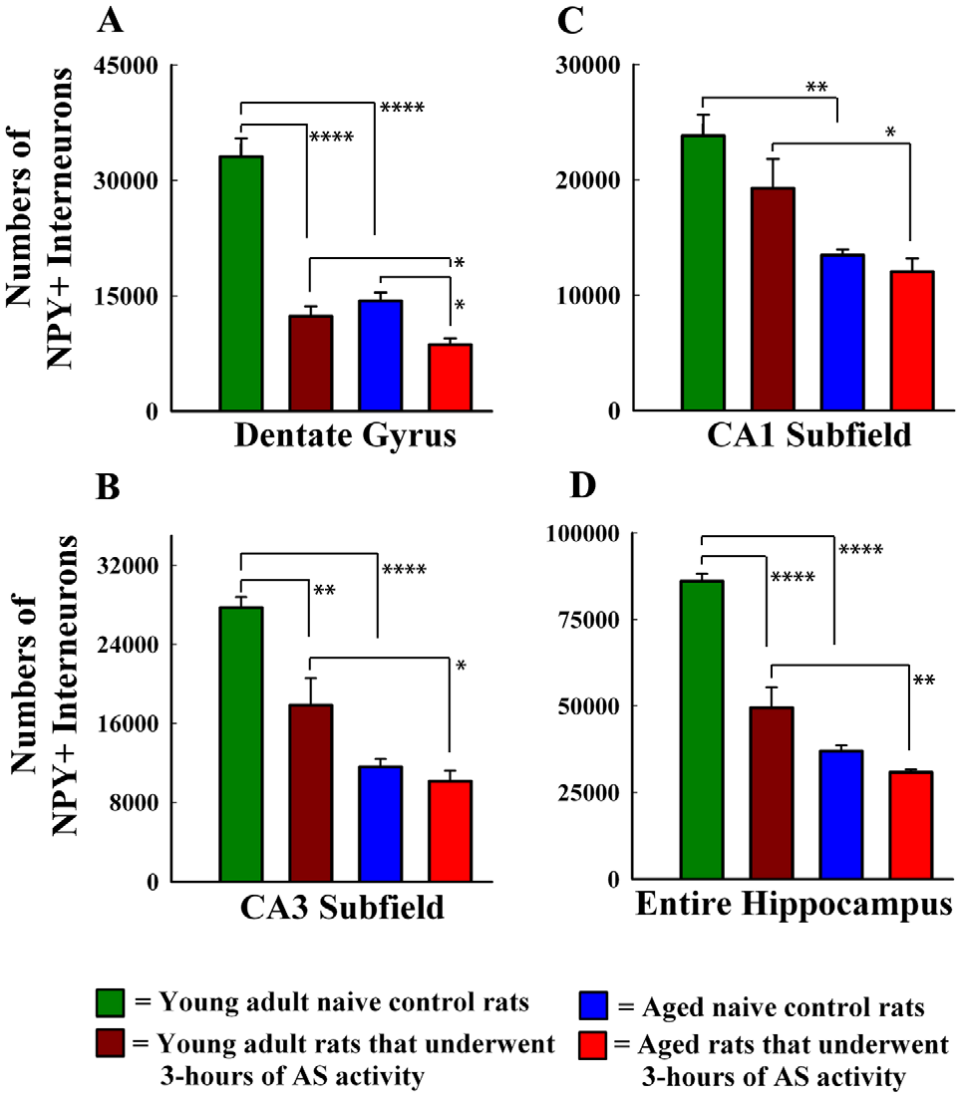
Comparison of the numbers of neuropeptide Y-positive (NPY+) interneurons in different regions of the hippocampus between the young adult naïve control rats, young adult rats that underwent three hours of acute seizure (AS) activity, aged naïve control rats, and aged rats that underwent three hours of AS activity. Two-way ANOVA analyses suggested significant main effects of age and AS activity for the dentate gyrus, the CA3 subfield and the entire hippocampus. Additionally, the interaction between age and AS activity was significant in these regions. In the CA1 subfield, there was a significant effect of age but not AS activity; there was also no interaction between age and AS activity (see “Results” section for details). Bonferroni post-tests revealed that: (i) the NPY+ interneurons in the dentate gyrus, the CA3 subfield, and the entire hippocampus of aged rats are less vulnerable to AS activity in comparison to their counterparts in young adult rats (A, B); and (ii) the NPY+ interneurons in the CA1 subfield are resistant to AS activity in both aged and young adult animals (C). Additionally note that, the residual numbers of NPY+ interneurons in different hippocampal regions of aged rats after AS activity are significantly lower than numbers in comparable regions of young adult rats that underwent similar AS activity (A–D), which is a consequence of reductions in the numbers of NPY+ interneurons with aging alone (A–D). *, p<0.05; **, p<0.01; ****, p<0.0001.

To analyze the effects of age and AS activity, and the potential role of interaction between age and AS activity in the overall reduction of NPY+ interneurons, two-way ANOVA analysis was performed for NPY+ neuron numbers from all four groups (young adult controls, young adults that underwent AS activity, aged controls, aged that underwent AS activity). With regard to the loss of NPY+ interneurons in the dentate gyrus, we found significant main effects of age (F = 54.6, p<0.0001; DFn = 1, DFd = 12) and AS activity (F = 75.9, p<0.0001; DFn = 1, DFd = 12). We also found a clear interaction between age and AS activity (F = 24.4; p<0.001; DFn = 1, DFd = 12). This was evidenced by a differential loss of NPY+ interneurons to AS activity between young adult rats (63% reduction, p<0.0001; Fig. 3 [A]) and aged rats (40% reduction, p<0.05; Fig. 3 [A]). However, as aging alone induced 57% reduction in NPY+ interneuron number (p<0.0001), the overall number of NPY+ neurons in the dentate gyrus of aged rats after AS activity was 30% less than the number in the dentate gyrus of young adult rats that underwent a similar AS activity (p<0.05; Fig. 3 [A]). The CA3 subfield also showed a similar trend pertaining to the loss of NPY+ interneurons. This was exemplified by significant main effects of age (F = 54.4, p<0.0001; DFn = 1, DFd = 12) and AS activity (F = 12.3, p<0.01; DFn = 1, DFd = 12) as well as an interaction between age and AS activity (F = 6.8, p<0.05; DFn = 1, DFd = 12; Fig. 3 [B]). The interaction was evidenced by a 36% reduction in the number of NPY+ interneurons in young adult rats (p<0.01; Fig. 3 [B]), and by no significant reduction in the number of NPY+ interneurons in aged rats (p>0.05; Fig. 3 [B]). However, as aging alone induced 58% reduction in NPY+ interneuron number (p<0.0001), the overall number of NPY+ interneurons in the CA3 subfield of aged rats after AS activity was 43% less than the number in the CA3 subfield of young adult rats after a similar AS activity (p<0.05; Fig. 3 [B].

In contrast to the above two regions, the CA1 subfield showed significant effects of age (F = 27.3, p<0.001; DFn = 1, DFd = 12) but not AS activity (F = 3.2, p>0.05; DFn = 1, DFd = 12). Furthermore, there was no interaction between age and AS activity (F = 0.9, p>0.05; Fig. 3 [C]) because AS activity induced no significant loss of NPY+ interneurons, neither in young adults (19% reduction, p>0.05; Fig. 3 [C]) nor in aged rats (11% reduction, p>0.05; Fig. 3 [C]). However, as aging alone induced 44% reduction (p<0.01) in the NPY+ interneuron number, the overall number of NPY+ neurons that persisted in the CA1 subfield of aged rats after AS activity was 38% less than the number in the CA1 subfield of young adult rats after a similar AS activity (p<0.05; Fig. 3 [C]). Comparison of the NPY+ interneuron numbers for the entire hippocampus between different groups revealed a trend that was comparable to what was seen for the dentate gyrus and the CA3 subfield (Fig. 3 [D]). There were significant main effects of age (F = 105.5, p<0.0001; DFn = 1, DFd = 12) and AS activity (F = 41.9, p<0.0001; DFn = 1, DFd = 12) as well as a clear interaction between age and AS activity (F = 21.4; p<0.001; Fig. 3 [D]). The interaction was evidenced by a greater loss of NPY+ interneurons in the hippocampus of young adult rats (42% reduction, p<0.0001; Fig. 3 [D]) than aged rats (17% reduction, p>0.05; Fig. 3 [D]) after comparable AS activity. However, as aging alone induced 57% reduction (p<0.0001) in the numbers of NPY+ interneurons, the overall numbers of NPY+ neurons that were surviving in the hippocampus of aged rats after AS activity was 48% less than the numbers in the hippocampus of young adult rats after similar AS activity (p<0.01; Fig. 3 [D]).

Thus, the NPY+ interneurons in the dentate gyrus and the CA3 subfield of aged rats were less vulnerable to AS activity in comparison to their counterparts in young adult rats. On the other hand, the NPY+ interneurons in the CA1 subfield were resistant to AS activity in both aged and young adult animals. Despite the above resistance, the residual numbers of NPY+ interneurons in different hippocampal regions of aged rats after AS activity were 30–43% lower than numbers in comparable regions of young adult rats that underwent similar AS activity. This discrepancy is clearly a consequence of reductions in the numbers of NPY+ interneurons with aging alone.

### Survival of PV+ hippocampal interneurons following three-hours of AS activity

**Figure 4:**
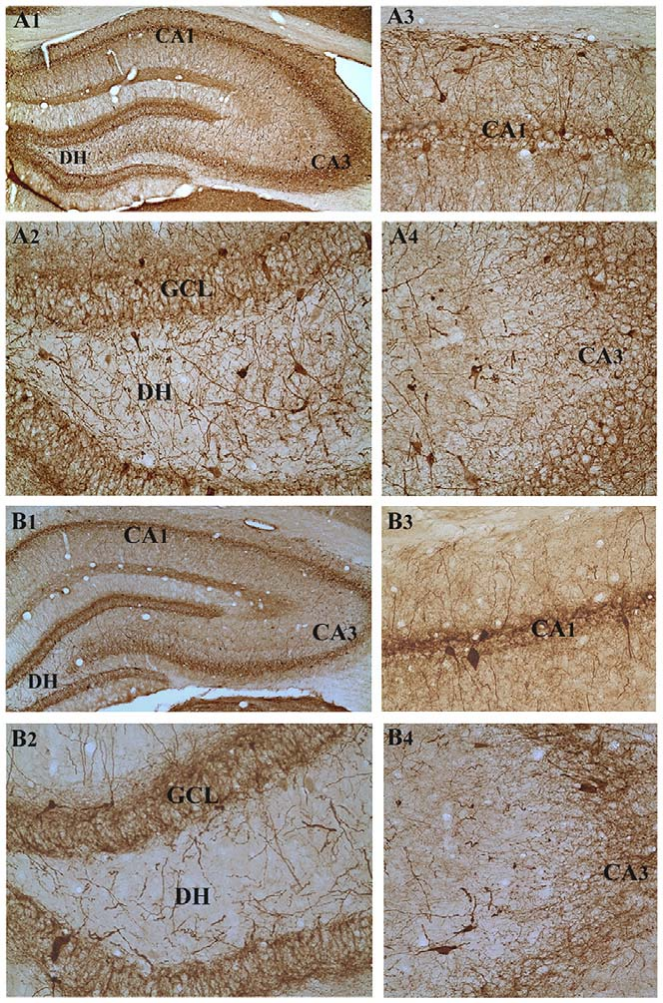
Distribution of parvalbumin-positive (PV+) interneurons in different subfields of the hippocampus of an intact young adult rat (A1) and a young adult rat that underwent three hours of acute seizure (AS) activity (B1). Figures A2–A4 show magnified views of the dentate gyrus, the CA1 subfield and the CA3 subfield from the figure A1. Figures B2–B4 show magnified views of the dentate gyrus, the CA1 subfield and the CA3 subfield from the figure B1. Note the paucity of PV+ cell bodies in different hippocampal regions after three hours of AS activity (B2–B4), in comparison to their counterparts in the intact young adult rat (A2–A4). Scale bar, A1 and B1 = 400 µm; A2–A4 and B2–B4 = 100 µm.

**Figure 5:**
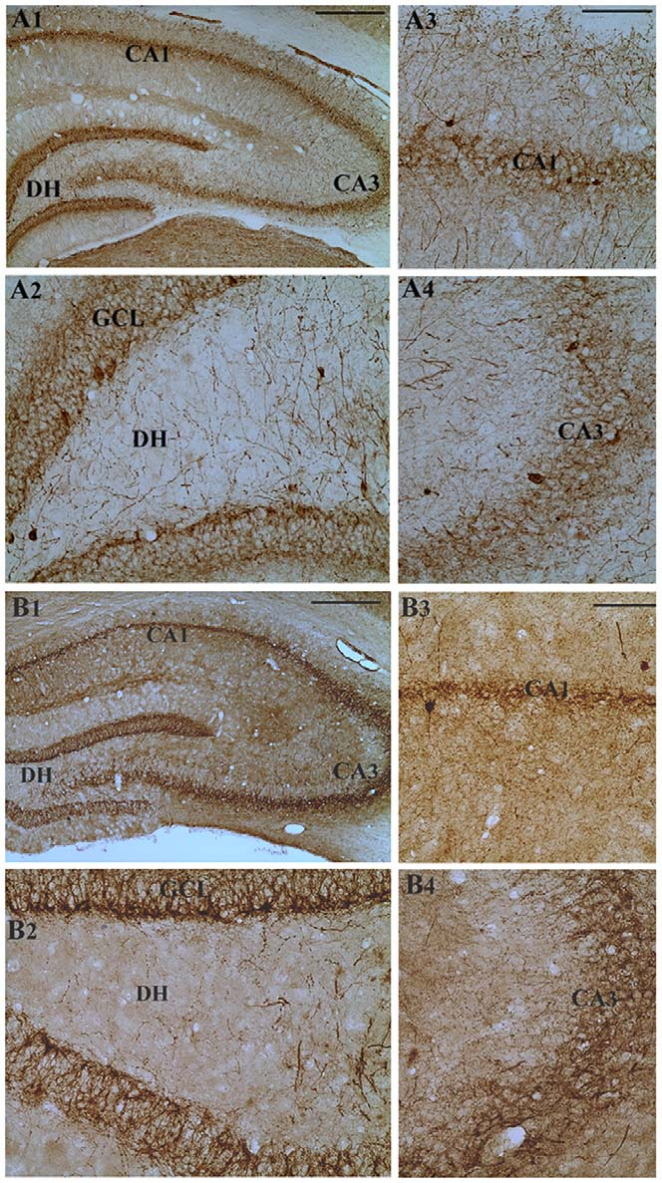
Distribution of parvalbumin-positive (PV+) interneurons in different subfields of the hippocampus of an intact aged rat (A1) and an aged rat that underwent three hours of acute seizure (AS) activity (B1). Figures A2–A4 show magnified views of the dentate gyrus, the CA1 subfield and the CA3 subfield from the figure A1. Figures B2–B4 show magnified views of the dentate gyrus, the CA1 subfield and the CA3 subfield from the figure B1. Note the paucity of PV+ soma in different hippocampal regions after three hours of AS activity (B2–B4), in comparison to their counterparts in the intact aged rat (A2–A4). Scale bar, A1 and B1 = 400 µm; A2–A4 and B2–B4 = 100 µm.

Immunohistochemical staining of sections with a PV antibody clearly visualized the pattern of distribution of PV+ interneurons in the dentate gyrus and the CA1 and CA3 subfields of both young and aged intact control rats (Figs 4 and 5 [A1]). In both age groups, the PV+ neurons were seen in the hilus and/or the granule cell layer of the dentate gyrus (Figs.4 and 5 [A2]), strata oriens and pyramidale of the CA1 subfield (Figs. 4 and 5 [A3]) and strata pyramidale and radiatum of the CA3 subfield (Figs. 4 and 5 [A4]). The density of PV+ interneurons in all subfields was greater in the young rats (Fig. 4 [A2–A4] than in aged rats (Fig. 5 [A2–A4]), which is consistent with our previous report [17]. In comparison to the age-matched intact hippocampi, three hours of AS activity markedly reduced the density of PV+ interneurons in different regions of both young and aged hippocampi (Figs. 4 and 5 [B1–B4]). However, the different hippocampal cell layers of both young and aged rats that underwent three hours of AS activity displayed a density of PV+ fibers (presumably axons; Figs. 4 and 5 [B2–B4]) that is either similar or greater than their counterparts in the age-matched intact rats (Figs. 4 and 5 [A2–A4). This may be due to a compensatory axonal sprouting of residual PV+ interneurons following the AS-induced loss of a greater proportion of PV+ interneurons in both age groups.

**Figure 6:**
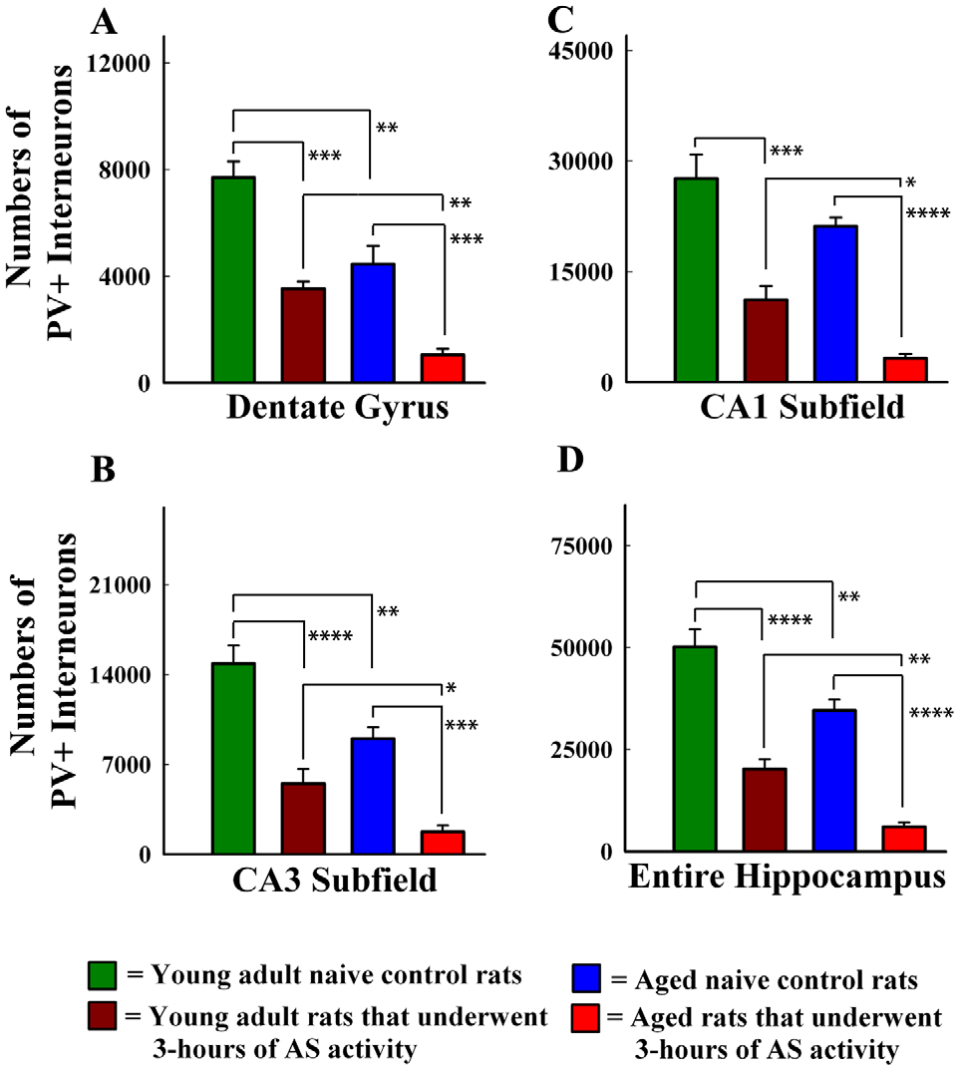
Comparison of the numbers of parvalbumin-positive (PV+) interneurons in different regions of the hippocampus between the young adult naïve control rats, young adult rats that underwent three hours of acute seizure (AS) activity, aged naïve control rats, and aged rats that underwent three hours of AS activity. A two-way ANOVA analysis suggested significant main effects of age and AS activity in all hippocampal regions but interaction between age and AS activity was absent (see “Results” section for details). Bonferroni post-tests revealed that the overall susceptibility of PV+ interneurons in different regions of the hippocampus to AS activity is similar between young adult and aged rats (A–D). Additionally, because of significant reductions in the numbers of PV+ interneurons with aging alone in most regions (A, B, D), the numbers of residual PV+ interneurons after AS activity in different hippocampal regions of aged rats are much lower than their counterparts in young adult rats after similar AS activity (A–D). *, p<0.05; **, p<0.01; ***, p<0.001; ****, p<0.0001.

To investigate the effects of age and AS activity, and the potential role of interaction between age and AS activity in the overall reduction of PV+ interneurons, two-way ANOVA analysis was performed for PV+ neuron numbers from all four groups. In all hippocampal regions, we found significant main effects of age (F = 13.1–33.6, p<0.01 to 0.0001; DFn = 1, DFd = 12) and AS activity (F = 58.8–74.4, p<00001; DFn = 1, DFd = 12). However, there was no interaction between age and AS activity (F = 0.1–0.6, p>0.05; DFn = 1, DFd = 12) for the loss of PV+ interneurons in any of the hippocampal regions because AS activity induced considerable reductions in the numbers of PV+ interneurons in both young adult and aged groups (Fig. 6). The reductions in young adult rats were 54% for the dentate gyrus (p<0.001; Fig. 6 [A]), 63% for the CA3 subfield (p<0.0001 Fig. 6 [B]), and 60% for the CA1 subfield (p<0.001) as well as for the entire hippocampus (p<0.0001; Fig. 6 [C]). The reductions in aged rats were 75% for the dentate gyrus (p<0.001; Fig. 6 [A]), 63% for the CA3 subfield (p<0.001; Fig. 6 [B), 85% for the CA1 subfield (p<0.0001; Fig. 6 [C]) and 82% for the entire hippocampus (p<0.0001; Fig. 6 [D]).

Thus, the susceptibility of PV+ interneurons in different regions of the hippocampus to AS activity is similar between the young adult and aged rats. However, because of significant reductions in the numbers of PV+ interneurons with aging alone (which comprised 42% for the dentate gyrus [p<0.01], 39% for the CA3 subfield [p<0.01], 23% for the CA1 subfield [p<0.01], and 31% for the entire hippocampus [p<0.01]), numbers of residual PV+ interneurons after AS activity in different hippocampal regions of aged rats were much lower than their counterparts in young adult rats that underwent similar AS activity. Overall, numbers of residual PV+ interneurons were 70% lower for the dentate gyrus, the CA1 subfield and the whole hippocampus (p<0.05 −0.01; Fig. 6 [A, C, D]), and 68% lower for the CA3 subfield (p<0.05; Fig. 6 [B]).

## Discussion

This study provides novel evidence of disparity in the susceptibility of two different subpopulations of interneurons to AS activity between the young adult hippocampus and the aged hippocampus. This was evidenced by the following observations. In comparison to the age-matched intact hippocampus, the overall diminution in the number of NPY+ interneurons after three-hour AS activity was substantial in the young adult hippocampus (42% reduction) but insignificant in the aged hippocampus (17% reduction). Additional subfield-specific quantification uncovered that AS activity induced loss of NPY+ interneurons in young adult rats was significant for the dentate gyrus and the CA3 subfield. In aged rats, it was significant only for the dentate gyrus. On the other hand, the overall decline in the number of PV+ interneurons after the three hours of AS activity was highly significant in both young adult hippocampus (60% reduction) and the aged hippocampus (82% reduction). Subfield-specific analyses revealed that AS activity induced loss of PV+ interneurons was considerable for all three regions of the hippocampus in both young adult and aged rats. Thus, in the aged hippocampus, the NPY+ interneurons are largely resistant to AS activity whereas PV+ interneurons are particularly vulnerable to AS activity. Interestingly, despite the above discrepancy in vulnerability between the two interneuron populations, residual numbers of both NPY+ and PV+ interneurons in the hippocampus of aged rats that underwent AS activity were much lower (48% lower for NPY+ interneurons and 70% lower for PV+ interneurons) than their counterparts in the hippocampus of young adult rats that underwent similar AS activity. This is mainly because of significant reductions in the numbers of NPY+ and PV+ interneurons with aging alone.

### Potential reasons for differential sensitivity of NPY+ and PV+ interneurons to AS activity in the aged hippocampus

A reduced vulnerability of NPY+ interneurons in the aged hippocampus is not due to a reduced number of seizures in the aged rats during the period of AS activity. This is because, seizure analyses during the three-hours of AS activity performed in these rats and reported earlier revealed no differences between the two age groups for the numbers of stage III or stage V seizures but increased numbers of stage IV seizures in aged rats [28]. Moreover, the discrepancy is also not due to a reduced hippocampal principal neuron loss in aged rats after AS activity because aged rats exhibited similar neurodegeneration and neuroinflammation as the young adult rats after AS activity except for the hippocampal CA1 region where aged rats exhibited much greater level of neurodegeneration [28]. On the other hand, a greater sensitivity of the PV+ interneurons to AS activity observed in both age groups could be because the PV+ interneurons being the fast-spiking interneurons have greater susceptibility to AS activity [39]. Another possibility is that the residual PV+ interneurons in the aged hippocampus have a greater level of afferent synaptic connectivity with principal hippocampal neurons (such as dentate granule cells, and CA1 and CA3 pyramidal neurons) than NPY+ interneurons. Furthermore, aging may be associated with differential changes in the intrinsic properties of hippocampal interneurons expressing NPY and PV such as a reduced excitability in NPY+ interneurons and a greater excitability in PV+ interneurons following afferent stimulation due to differential changes in glutamate or other receptors that promote excitatory neurotransmission in these interneurons. Additionally, it may be that the firing of principal neurons in the aged hippocampus during AS activity is not strong enough to cause widespread degeneration of NPY+ interneuron numbers but adequate for inducing degeneration of fast-spiking PV+ interneurons. Indeed, a previous study showed that while both young and aged rats exhibited an increase in the EEG power during the AS activity, visual inspection and spectral analysis revealed a reduction of the faster frequencies in the EEGs of aged animals despite a shorter latency to stage V seizures in comparison to young rats [40]. It is conceivable that a reduced EEG activity in aged animals is sufficient for inducing degeneration of significant numbers of hippocampal principal neurons and PV+ interneurons but not NPY+ interneurons. Further studies are required to recognize the contribution of above possibilities to the relative resistance of NPY+ interneurons in the aged hippocampus to AS activity.

### Implications of reduced numbers of NPY+ interneurons in the aged hippocampus after AS activity

In the hippocampus, the NPY+ inhibitory GABA-ergic interneurons are mainly distributed in the dentate hilus and the CA1 and CA3 strata oriens and pyramidale [12], [38], [41]. In the dentate gyrus, the axons of these interneurons synapse on the excitatory dentate granule cells. The NPY is essential for various functions in the hippocampus. First, the NPY+ interneurons in the hippocampus play vital roles in inhibiting the activity of hippocampal circuitry by consistently hyperpolarizing and reducing the spike frequency of excitatory neurons [33]. Previous studies also indicate that the NPY modulates the excitatory synaptic neurotransmission in the hippocampus by inhibiting the glutamate release on to principal hippocampal neurons [42], [43]. Second, the NPY is an anticonvulsant protein. This is supported by many previous observations, which comprise an increased hippocampal NPY expression in response to status epilepticus in young adult rats as a compensatory means to reduce seizures [32], [44], [45], seizure activity in response to reduced levels of NPY [45], and a reduced epileptiform-like activity with the administration of NPY in animal models of epilepsy [46]–[49]. Third, NPY has a role in the hippocampal neurogenesis and functions such as learning, memory and mood, as it enhances the proliferation of neural stem cells and augments the mitogenic effect of the fibroblast growth factor-2 on neural stem cells [31], [32], [50].

From the above, it appears that considerably decreased number of NPY+ interneurons observed in both young adult and aged hippocampi after AS activity plays a role in the progression of AS activity into chronic epilepsy and cognitive dysfunction. However, as the overall number of residual NPY+ neurons in the aged hippocampus after AS activity is 48% fewer than the young adult hippocampus after similar AS activity, the implications of reduced NPY levels on hippocampal functions are likely much greater in aged animals. These may include significantly decreased inhibition of hippocampal excitatory neurons resulting in widespread epileptiform activity and spontaneous seizures, and vastly decreased hippocampal neurogenesis and severely impaired hippocampal-dependent learning and memory function. Indeed, our earlier report has shown that aged rats exhibit much greater propensity for developing TLE characterized by spontaneous recurrent seizures after 3 hours of AS activity than young adult rats undergoing similar AS activity [28]. Furthermore, the overall intensity and severity of spontaneous recurrent seizures in the chronic phase after AS activity were much greater in aged rats than in young adult rats [28]. Additionally, aged rats that underwent 3 hours of AS activity were incapable of learning a spatial task in a water maze test whereas young adult rats that underwent similar AS activity exhibited capacity for spatial learning but had memory dysfunction [28].

### Implications of substantially reduced PV+ interneuron numbers in the aged hippocampus after AS activity

In the hippocampus, PV+ inhibitory GABA-ergic interneurons are typically observed in the dentate granule cell layer (as basket cells), and the strata oriens and pyramidale of CA1–CA3 subfields [51], [52]. The calcium binding protein PV+ interneurons have crucial functions in the hippocampus. First, these fast-spiking interneurons are a prime source of perisomatic inhibition onto hippocampal pyramidal neurons in the CA1–CA3 regions [12]. Second, the activity of PV+ interneurons is essential for maintaining the working memory function of the hippocampus, as selective ablation of PV+ interneurons in the CA1 subfield induces spatial working memory impairments [53]. Third, the activity of PV+ interneurons is necessary for synchronizing the hippocampal pyramidal neurons during network oscillations [34]. Because hippocampal network oscillations coordinate the firing of large neuronal populations at different time scales and maintain conditions for adaptive operation of networks during data encoding, processing and storage, the PV+ interneurons have functional relevance for contributing to cognitive processes handled by the hippocampus [54]. Fourth, reductions in the PV+ interneuron subpopulation can be a key factor in the epileptogenic process, as PV deficiency affects network properties resulting in an increased susceptibility to seizures [35], [36]. Our results show that, three hours of AS activity results in a substantial loss of PV+ interneurons in all regions of the hippocampus in both young adult and aged rats. The findings in young adult rats are consistent with the earlier reports on epileptic hippocampal tissues from TLE patients and animal models of TLE [55]–[58]. Previous studies show that seizure-induced loss of PV+ interneurons in the dentate gyrus can considerably reduce the inhibition of granule cells [59]–[63]. Because of a substantial loss suffered during the process of aging and a relatively greater loss in response to AS activity, the aged rats that underwent AS activity exhibit 70% lower PV+ interneurons than the young adult rats that underwent similar AS activity. From this perspective, it is likely that extensively decreased PV+ interneuron numbers in the aged hippocampus after AS activity contribute significantly to impairments in the various hippocampal functions described earlier [28].

### Conclusion

This study demonstrates that the vulnerability of NPY+ and PV+ interneurons to three hours of AS activity in the aged hippocampus is distinct. While the NPY+ interneurons in the aged hippocampus were relatively resistant, the PV+ interneurons were highly susceptible to AS activity. Nonetheless, the residual numbers of both NPY+ and PV+ interneurons in the hippocampus of aged rats that underwent AS activity were much lower, in comparison to their counterparts in the hippocampus of young adult rats that underwent similar AS activity. This is mainly because of significant reductions in the numbers of NPY+ and PV+ interneurons with aging alone. Overall, these results underscore that AS activity-induced TLE in old age is linked with far fewer hippocampal NPY+ and PV+ interneuron numbers than AS-induced TLE in the young adult age. This discrepancy likely underlies the much severe spontaneous seizures and cognitive dysfunction observed in the aged population after AS activity [28].

## Materials and Methods

Four groups of Fischer 344 (F344) rats obtained from the National Institutes of Aging colony at Harlan Sprague-Dawley (Indianapolis, IN) were used in this study. The groups comprised intact young adult rats (4–5 months old; n = 5), intact aged rats (22 months old, n = 5), young adult rats receiving graded intraperitoneal injections of KA (n = 6), and aged rats receiving graded intraperitoneal injections of KA (n = 7). The animals were housed in an environmentally controlled room with a 12∶12-hr light-dark cycle and were given food and water ad libitum. All experiments were performed as per the animal protocol (VA protocol #1105-006), approved by the animal studies subcommittee of the Durham Veterans Affairs Medical Center.

### Induction of AS activity and SE in young adult and aged rats

A detailed methodology for the induction of AS activity and SE in young adult and aged rats is described in our recent report [28]. In brief, rats received graded intraperitoneal injections of kainic acid (KA; 3mg/kg) every hour until they developed SE. The KA injections were terminated when rats displayed either a state of continuous stage IV seizures characterized by bilateral forelimb clonus with signs of rearing, or a first stage V seizure typified by bilateral forelimb clonus with rearing and falling followed by continuous stages III-V seizures for over 10 minutes. While young adult rats required an average of four hourly injections of KA for eliciting SE, the induction of SE in aged rats required an average of two hourly injections of KA [28]. Both groups of rats were allowed to have multiple stages III-V seizures after the onset of SE. However, after three hours of AS activity, all motor seizures were terminated with a single injection of diazepam (5 mg/Kg body weight [bw]). Despite the differing doses of KA required for induction of SE and AS activity, the severity of AS activity in terms of stages III-V seizures during a three-hour period were largely comparable between the two age groups [28].

### Animal perfusions and tissue processing

Twelve days after the three hours of AS activity, surviving rats from both groups (n = 5/group) were fatally anesthetized with isoflurane. Following this, the rats were perfused transcardially with 4% paraformaldehyde solution. The rats from control groups (intact young adult and intact aged groups) were also similarly perfused. The brains were dissected out, post-fixed in 4% paraformaldehyde for 16 hours at 4°C and cryoprotected in 30% sucrose solution in phosphate buffer (PB). Thirty-micrometer thick cryostat sections were cut coronally through the entire septo-temporal axis of the hippocampus and collected serially in 24-well plates filled with PB. Every 20th section through the entire hippocampus was then selected in each of the animals and processed for NPY immunohistochemistry, which visualized the NPY+ interneurons in different subfields of the hippocampus. A second set of sections (every 20th) were processed for PV immunohistochemistry for visualization of PV+ interneurons in different regions of the hippocampus.

### NPY immunohistochemistry

A detailed methodology for NPY immunohistochemistry is described elsewhere [37], [38]. Briefly, the sections were treated with 0.1 M Tris buffer (TB) containing 1% hydrogen peroxide for 30 minutes, washed in TB, treated consecutively with TB containing 0.1% Triton X-100 (Tris A; 10 minutes) and TB containing 0.1% Triton X-100 and 0.005% bovine serum albumin (Tris B) for 10 minutes each. Following this, the sections were incubated in a blocking solution containing 10% normal goat serum in Tris B for 45 minutes. Sections were washed again in Tris A and Tris B and incubated in rabbit anti-NPY antibody (1∶1000, Peninsula laboratories, San Carlos, CA.) for 48 hours at 4°C, washed consecutively in Tris A and Tris B solutions, and incubated in biotinylated goat anti-rabbit IgG (1∶1000; Vector) for 45 minutes. Following this, sections were washed successively in Tris A and Tris D (0.5 M TB containing 0.1% Triton X-100 and 0.005% bovine serum albumin), and incubated in the avidin-biotin complex (ABC; Vector) solution diluted in Tris D (1∶1000) for 60 minutes. The tissue-bound peroxidase was then developed using vector gray (Vector) as a chromogen. The sections were mounted on gelatin coated slides, dehydrated, cleared, and cover slipped with DPX.

### PV immunohistochemistry

A detailed methodology for PV immunohistochemistry is described in our earlier report [17]. In brief, the sections were treated with PBS solution containing 20% methanol and 3% hydrogen peroxide for 20 minutes and then rinsed thrice in PBS. The sections were next blocked with 10% normal horse serum in PBS containing 0.1% Triton-X 100, and incubated overnight in a mouse anti-parvalbumin antibody solution (1∶2000 in PBS, Sigma). Following this, the sections were washed in PBS, treated with the biotinylated anti-mouse IgG solution (Vector) for 60 minutes, washed in PBS, and incubated with the ABC reagent (Vector) for 60 minutes. The peroxidase reaction was developed using the diaminobenzidine (DAB) as a chromogen (Vector). The sections were mounted on gelatin coated slides, dehydrated, cleared, and cover slipped with DPX.

### Measurement of NPY+ and PV+ interneurons in the dentate gyrus and CA1 & CA3 subfields

The numbers of NPY+ and PV+ interneurons were measured for the dentate gyrus and the CA1 and CA3 subfields of the hippocampus in every 20th section through the entire septo-temporal axis of the hippocampus, in each animal belonging to the four groups (n = 5/group). An optical fractionator method using the StereoInvestigator system (Microbrightfield Inc) was employed for counting of NPY+ and PV+ interneurons. The StereoInvestigator system consisted of a color digital video camera (Optronics Inc) interfaced with a Nikon E600 microscope.

### Optical fractionator counting method

A detailed methodology is described in our recent report [28]. Cells that are positive for NPY/PV in each of the three selected regions were counted from 50–500 frames chosen via a systematic random sampling procedure in every 20th section using a 100X oil immersion lens. A counting frame measuring 40×40 µm was used for all three regions (the dentate gyrus and the CA1 and CA3 subfields). For counting of cells in each section, the contours of different regions (the dentate gyrus and the CA1 and CA3 subfields) were first delineated using the tracing function of the StereoInvestigator. Following this, the optical fractionator unit was activated and the number and location of counting frames and the counting depth for each section was determined by entering parameters such as the grid size, the thickness of the top guard zone (4 µm) and the optical dissector height (8 µm). A computer controlled motorized stage then allowed the section to be analyzed at each of the counting frame locations. All NPY+/PV+ interneurons that were present within the 8 µm section depths in each location were counted. The above procedure was repeated for all serial sections. An option in the Stereo Investigator program allowed the experimenter to remain unaware of the running cell count totals until all sections for an animal were completed. The StereoInvestigator program then calculated the total number of NPY+/PV+ interneurons per each region by utilizing the optical fractionator formula, N = 1/ssf.1/asf.1/hsf.EQ-. The abbreviation ssf represents the section sampling fraction, which was 20 in this study, as every 20th section was sampled. asf symbolizes the area sampling fraction, which was calculated by dividing the area sampled with the total area of the respective subfield (i.e. the sum of subfield areas sampled in every 20th section). hsf stands for the height sampling fraction, which was calculated by dividing the height sampled (i.e. 8 µm in this study) with the section thickness at the time of analysis (i.e. 20 µm in intact control animals and 15 µm in animals that underwent 3 hours of AS activity). EQ- denotes the total count of particles sampled for the entire subfield.

### Data analyses

The values for NPY+ and PV+ interneurons were calculated separately for each region in every animal before calculating the means ± standard errors (S.E.M.) for the four groups. The NPY+/PV+ interneuron counts in different regions of intact young adult rats (n = 5), young adult rats that underwent 3-hours of AS activity (n = 5), intact aged rats (n = 5), and aged rats that underwent 3-hours of AS activity (n = 5) were compared to determine the main significant effects of age and AS activity and the potential interaction between age and AS activity on the extent of interneuron loss. For this, values from the above four groups were analyzed using two-way ANOVA with Bonferroni post-tests. The differences were considered significant if the p values were found to be less than 0.05. Specifically, we examined whether the effects of 3-hours of AS activity on the survival of NPY+ and PV+ interneurons in different regions of the hippocampus differ between the young adult and aged groups.
